# Emergency Room Evaluation and Recommendations and Risk Screening of Incident Major Neurocognitive Disorders in Older Females: Results of an Observational Population-Based Cohort Study

**DOI:** 10.3389/fnagi.2022.912477

**Published:** 2022-07-22

**Authors:** Olivier Beauchet, Jacqueline Matskiv, Cyrille P. Launay, Yves Rolland, Anne-Marie Schott, Gilles Allali

**Affiliations:** ^1^Department of Medicine and Geriatrics, University of Montreal, Montreal, QC, Canada; ^2^Research Centre of the Geriatric University Institute of Montreal, Montreal, QC, Canada; ^3^Division of Geriatric Medicine, Department of Medicine, Sir Mortimer B. Davis Jewish General Hospital and Lady Davis Institute for Medical Research, McGill University, Montreal, QC, Canada; ^4^Lee Kong Chian School of Medicine, Nanyang Technological University, Singapore, Singapore; ^5^Department of Geriatric, Toulouse University Hospital, Toulouse, France; ^6^Université Claude Bernard Lyon1, Unité INSERM 1290 RESHAPE, Hospices Civils de Lyon, Pôle de Santé Publique, Lyon, France; ^7^Leenaards Memory Center, Lausanne University Hospital and University of Lausanne, Lausanne, Switzerland

**Keywords:** older adults, epidemiology, cohort study, dementia, incidence

## Abstract

**Background:**

“Emergency Room Evaluation and Recommendations” (ER^2^) risk levels (i.e., low, moderate and high) may be used to screen for major neurocognitive disorders (MNCD) in older emergency department users, as a high ER^2^ risk level is associated with MNCD diagnosis. This study aims to examine the association of ER^2^ risk levels with incident MNCD in community-dwelling older adults.

**Methods:**

A total of 709 participants of the EPIDémiologie de l’OStéoporose (EPIDOS) study—an observational population-based cohort study—were recruited in Toulouse (France). ER^2^ low, moderate and high risk levels were determined at baseline. Incident MNCD and their type (i.e., Alzheimer’s disease (AD) vs. non-AD) were diagnosed after a 7-year follow-up period.

**Results:**

The overall incidence of MNCD was 29.1%. A low ER^2^ risk level was associated with low incidence of MNCD [Hazard ratio (HR) = 0.71 with *P* = 0.018] and AD (HR = 0.56 with *P* = 0.003), whereas a high risk level, both individually and when combined with a moderate risk level, was associated with high incidence of MNCD (HR ≥ 1.40 with *P* ≤0.018) and AD (HR ≥ 1.80 with *P* ≤ 0.003). No association was found with incident non-AD.

**Conclusion:**

ER^2^ risk levels were positively associated with incident MNCD in EPIDOS participants, suggesting that ER^2^ may be used for risk screening of MNCD in the older population.

## Background

Major neurocognitive disorders (MNCD) are highly prevalent in the older population (Alzheimer’s Association, 2021). Even if age-specific incidence rates are lower than previous decades, the number of people with MNCD is still rising (Livingston et al., 2020). Today, 55 million people live with MNCD worldwide, and this figure is expected to triple over the next decade (Alzheimer’s Association, 2021). MNCD cause adverse outcomes in physical, psychological and social domains for patients, as well as their caregivers and society at large (Livingston et al., 2020; Alzheimer’s Association, 2021). Care strategies for MNCD have gradually shifted from curative to preventive interventions which emphasize the role of modifiable risk factors (MRFs) (Curran et al., 2021). But for prevention to occur, those at risk must be identified (Curran et al., 2021). Screening people at risk of MNCD is the first step of an effective strategy to delay or reduce the incidence of MNCD (Livingston et al., 2020; Curran et al., 2021).

“Emergency Room Evaluation and Recommendations” (ER^2^) is a simple clinical assessment tool stratifying risks of adverse outcomes into three levels (i.e., low, moderate and high) in older emergency department (ED) users (Launay et al., 2021b). The prevalence of MNCD in older ED users is around 30%. This population is more prone to a high risk of adverse outcomes compared to ED visitors without MNCD, in part because they are underdiagnosed (Launay et al., 2021b; Beauchet et al., 2022). Recently, we showed that a high ER^2^ risk level successfully screened older ED users with MNCD upon their arrival to the ED (Beauchet et al., 2022). Generally, MNCD and risk of MNCD remain under-screened in the community (Boustani et al., 2003; Haubois et al., 2011). The narrow window for assessment may in part account for this (Boustani et al., 2003). There is a need for simple and efficient MNCD screening tests in community-dwelling older adults. ER^2^ is a simple clinical tool which can be easily performed in primary care. An association between ER^2^ risk levels and incident MNCD has not yet been reported. We hypothesized that ER^2^ risk levels could be used to screen community-dwelling older adults at risk of incident MNCD. Given that Alzheimer’s disease (AD) is the most common cause of MNCD in the older population, the probability of detecting individuals at risk of AD with a test like the ER^2^ may be greater when compared to non-AD detection (Livingston et al., 2020; Alzheimer’s Association, 2021). This study thus aims to examine the association of ER^2^ risk levels with incident MNCD, with an emphasis on AD, in this population.

## Materials and Methods

### Design

**FIGURE 1:**
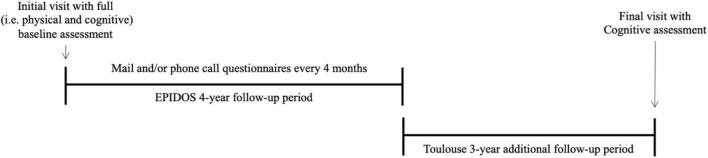
Overview of the follow-up period.

We had the opportunity to use the database of the “EPIDémiologie de l’OStéoporose” (EPIDOS) study, which is an observational population-based cohort study (Dargent-Molina et al., 1996). This study was originally designed to examine risk factors for hip fracture in older French women. Participants recruited in Toulouse (France) between January 1992 and January 1994 were assessed by mail and/or phone questionnaires every 4 months, over an initial follow-up period of 4 years (like the other EPIDOS participants). Following this initial period (i.e., between 1996 and 1998), this subset of participants were invited to take part in an additional 3-year follow-up. Only one visit was planned at the end of this second follow-up period (i.e., between 1999 and 2002). This final visit took place at the University Hospital of Toulouse or at the participants’ home. Thus, the Toulouse EPIDOS participants had two exhaustive assessments: at baseline, when they were enrolled in the study, and at the end of the 7-year follow-up (Figure 1).

### Population

7,598 women aged ≥ 75 and living in one of five French cities (Amiens, Lyon, Montpellier, Paris and Toulouse) were recruited at baseline. 1,462 (19.2%) participants among this initial EPIDOS set were recruited in Toulouse and agreed to take part in an additional 3 years of follow-up. From this subset, we excluded participants with a suspicion of MNCD at the initial baseline assessment of the EPIDOS cohort (i.e., 1992–1994) using the threshold value of ≥ 3 incorrect answers on the Short Portable Mental Status Questionnaire (SPMSQ), as well as those without information on their cognitive status at the end of follow-up (i.e., no MNCD vs. MNCD and its etiology coded as Alzheimer’s Disease (AD) vs. non-AD) (Pfeiffer, 1975). At the end of the 7-year follow-up, the cognitive status of 714 women was known (48.8% of the initial Toulouse cohort). Among this subset, 5 participants were excluded because information about ER^2^ was missing. Finally, 709 (48.5%) participants were selected for the study.

### Baseline Assessment

Information on age, living situation (in residence or not, with or without someone), frequency of contacts with others over the past week, education level, number of drugs taken daily, weight (in kg), height (in cm), regular physical activity (i.e., ≥ 1 h a week during the past month), use of a walking aid regardless of its type, history of falls in the past 6 months, and inability to name the date (an item of SPMSQ) were recorded during a standardized face-to-face baseline assessment (Pfeiffer, 1975). Body mass index (BMI) was calculated and overweight and/or obesity were considered present if BMI was ≥ 25 kg/m^2^. Polypharmacy was defined as ≥ 5 drugs taken daily. A high education level was defined as high school or above.

### Emergency Room Evaluation and Recommendations Risk Stratification

Six close-ended questions (i.e., yes vs. no) comprise the ER^2^ (Launay et al., 2021b; Beauchet et al., 2022). They include age (≥85), sex (i.e., male vs. female), polypharmacy, use of formal (i.e., healthcare and/or social services) and/or informal (i.e., family and/or friends) home support, use of a walking aid regardless of type and/or history of falls in the past 6 months, temporal disorientation (i.e., inability to name the current month and/or year). For the present study, two ER^2^ items adapted. First, “use of formal and/or informal home support” was changed to “lives with someone and/or had contact with someone over the past week.” We made this changed because the targeted population in the EPIDOS study was community-dwelling older adults in a relatively good health condition at baseline, which differs from the older populations used in studies for the development of ER^2^. Formal or informal home support are not only a criterion of functional decline but also of the individual’s social network (Launay et al., 2021b; Beauchet et al., 2022). Thus, we made the decision to use this component for the adaptation of this ER^2^ criterion. Second, temporal disorientation was defined as the inability to name the date (an item of SPMSQ). The ER^2^ item used in previous studies was an inability to name the month and/or the year. This level of information was not accessible in the EPIDOS database. Thus, a surrogate measure was to consider the date. A score of 5 points was assigned to items “use of a walking aid” and “temporal disorientation,” while a score of one point was assigned to the other items as defined and validated in previous studies (Launay et al., 2021a,b; Beauchet et al., 2022). The ER^2^ score ranged from 0 (lowest risk) to 14 (highest risk) and stratified risk of adverse outcomes into low (score 0–3), moderate (score 4–5) and high (score ≥6) risk (Launay et al., 2021a,b; Beauchet et al., 2022).

### Definition of Major Neurocognitive Disorders

A cognitive assessment was performed at the seventh year of follow-up using standardized tests including the SPMSQ (Pfeiffer, 1975), the Mini Mental State Examination (MMSE) (Folstein et al., 1975), and the Grober and Buschke test (i.e., Free and cued selective reminding test) (Grober et al., 1988). The SPMSQ and MMSE assess global cognitive functioning. The SPMSQ score ranges from 0 (no cognitive impairment) to 10 (severe cognitive impairment). A score between 0 and 2 means no cognitive impairment. The MMSE score ranges from 0 (severe cognitive impairment) to 30 (no cognitive impairment). A MMSE score below 26 means cognitive impairment. The Grober and Buschke test is a verbal episodic memory test which controls for attention and acquisition deficit, by providing category cues in the learning process. Its score ranges from 0 (severe memory impairment) to 48 (no impairment), with a total recall cut-score of ≤ 46/48. In addition, results of brain imaging reports and/or the images themselves were reviewed to contribute to the diagnosis of MNCD subtypes [i.e., cortical and subcortical atrophy vs. brain abnormalities like microvascular ischemic lesion, infarcts, intracerebral lesions (tumors or hematomas] and enlarged ventricles]. The results of all tests were analyzed by a geriatrician and a neurologist in a double-blind manner. DSM-IV criteria were used for the diagnosis of MNCD (American Psychiatric Association, 1994). AD was diagnosed using the criteria of the NINCDS-ADRDA Work Group (McKhann et al., 1984; Tucker, 1999; Hogervorst et al., 2000). Participants who satisfied DSM-IV criteria but not NINCDS-ADRDA criteria were classified with a diagnosis of non-AD. Participants were separated in 2 groups: No MNCD and MNCD with two subsets (i.e., AD and non-AD).

### Standard Protocol Approval and Patient Consents

This study was conducted in accordance with the ethical standards set forth in the Helsinki Declaration (1983). The Research Ethics Board (REB) of Toulouse University Hospital approved the EPIDOS protocol. Written informed consent for research was obtained for all recruited EPIDOS participants.

### Statistics

Means, SD, and percentages described the participants’ characteristics. Participants were divided according to their cognitive status: with or without MNCD. Group comparisons were performed using unpaired *t*-tests or Chi square tests, as appropriate. Cox regressions were performed to examine the association of ER^2^ risks (independent variable; separated model for each risk level) and incident MNCD (all categories, non-AD and AD; dependent variable; separated model for each type of MNCD). All models are adjusted by place of living, high education level, abnormal body mass index (i.e., ≥ 25 kg/m^2^) and regular physical activity. *P*-values < 0.05 were considered statistically significant. All statistics were performed using SPSS (version 28.0; SPSS, Inc., Chicago, IL).

## Results

The incidence of MNCD was 29.1% (Table 1). Participants who developed MNCD were older (*P* ≤0.001), lived more frequently in residence (*P* = 0.005), had a lower education level (*P* ≤ 0.001) and lower physical activity levels (*P* = 0.035), and took more medications (*P* = 0.017) compared to those without MNCD at the end of follow-up. The mean ER^2^ score at baseline was higher in participants who had incident MNCD than in those who stayed cognitively healthy (*P* ≤ 0.001). The prevalence of older participants (i.e., > 85) (*P* = 0.002), home support (*P* = 0.010) and inability to name the date (*P* ≤ 0.001) were higher in the group with incident MNCD compared to participants who stayed cognitively healthy over the 7 years. The prevalence of participants with a low ER^2^ risk level was lower in the group that developed MNCD than in those who stayed cognitively healthy (*P* = 0.001), whereas there were more participants with a high ER^2^ risk level in the group with incident MNCD when compared to their MNCD-free counterparts (*P* ≤ 0.001).

**TABLE 1:** Comparisons of participant’s baseline characteristics and incident major neurocognitive disorders according to their cognitive status at the end of the 7-year follow-up (*n* = 709).

	Major neurocognitive disorders		*P*-value*
		
	No (*n* = 503)	Yes (*n* = 206)	
Age, mean ± SD (year)	79.3 ± 3.5	81.2 ± 3.8	**≤0.001**
Living in residence, n (%)	44 (8.7)	33 (16.0)	**0.005**
High education level^†^, n (%)	236 (46.9)	63 (30.6)	**≤0.001**
Number of drugs taken daily, mean ± SD	4.9 ± 2.8	5.4 ± 3.0	**0.017**
**Body mass index (kg/m^2^)**			
Mean ± SD	25.1 ± 3.8	24.9 ± 4.2	0.547
≥25	233 (46.3)	91 (44.2)	0.587
Regular physical activity^‡^	224 (44.5)	73 (35.4)	**0.035**
**ER^2^**			
Mean ± SD score (/14)	3.5 ± 3.0	4.8 ± 3.5	**≤0.001**
Items, n (%)			
Age ≥ 85	38 (7.6)	31 (15.0)	**0.002**
Polypharmacy^#^	265 (52.7)	123 (59.7)	0.088
Home support¶	292 (58.1)	141 (68.4)	**0.010**
Use of walking aide and/or history of fall in the past 6 months	151 (30.0)	76 (36.9)	0.075
Inability to name day’s date	83 (16.5)	64 (31.1)	**≤0.001**
Level of risk**, n (%)			
Low	292 (58.1)	92 (44.7)	**0.001**
Moderate	36 (7.2)	9 (4.4)	0.167
High	175 (34.8)	105 (51.0)	**≤0.001**
Non-Alzheimer disease	–	96 (46.6)	–
Alzheimer disease	–	110 (53.4)	–

*ER^2^, Emergency room evaluation and recommendations; SD, Standard deviation.*

*Comparisons based on unpaired-t-test or Chi square test, as appropriate.

*^†^High school and greater.*

*^‡^At least one recreational physical activity (walking, gymnastics, cycling, swimming or gardening) for at least 1 h a week over the past month or more.*

*^#^Number of drugs taken daily = 5.*

*^¶^Living with someone and/or having contact with someone over the past week.*

^**^ER^2^ score ranged from 0 to 14 with low risk (score 0–3), moderate risk (score 4–5) and high risk s(core = 6; P-value significant (i.e., P < 0.05) indicated in bold.

Cox regressions revealed that a low ER^2^ risk level was significantly associated with low incidence of MNCD and AD [Hazard ratio (HR) = 0.71 with *P* = 0.018 and HR = 0.56 with *P* = 0.003, respectively], whereas the ER^2^ high risk level, both individually and merged with the moderate level, was significantly associated with high incidence of MNCD and AD (HR ≥ 1.40 with *P* ≤ 0.018 and HR ≥1.80 with *P* ≤ 0.003, respectively Table 2). When participants with a low ER^2^ risk level were used as the reference group, only the ER^2^ high risk level was associated with high incidence of MNCD (HR = 1.50 with *P* = 0.006) and AD (HR = 1.94 with *P* = 0.001).

**TABLE 2:** Cox regressions showing the association between ER^2^ risk levels (independent variable; separated model for each risk level) and incident major neurocognitive disorders (all categories, non-Alzheimer’s disease, Alzheimer’s disease; dependent variable; separated model for each variable) in EPIDOS participants (*n* = 709).

ER^2^ scores and risk levels	Major neurocognitive disorders								
	
	All categories			Non-Alzheimer’s disease			Alzheimer’s disease		
			
	HR	[95% CI]	*P*-value	HR	[95% CI]	*P*-value	HR	[95% CI]	*P*-value
Score 0–3 (low risk)	0.71	[0.54;0.94]	**0.018**	0.95	[0.63;1.42]	0.784	0.56	[0.38;0.82]	**0.003**
Score 4–5 (moderate risk)	0.64	[0.32;1.31]	0.643	0.66	[0.24;1.81]	0.424	0.63	[0.23;1.71]	0.360
Score = 6 (high risk)	1.53	[1.16;2.02]	**0.003**	1.15	[0.76;1.74]	0.499	1.96	[1.34;2.89]	**0.001**
Score = 4 (moderate and high risk combined)	1.40	[1.06;1.85]	**0.018**	1.06	[0.71;1.59]	0.784	1.80	[1.22;2.67]	**0.003**
**ER^2^ level of risk:**									
Low*	Ref.			Ref.			Ref.		
Moderate^†^	0.78	[0.38;1.61]	0.497	0.70	[0.25;1.94]	0.696	0.87	[0.31;2.45]	0.797
High^‡^	1.50	[1.13;1.99]	**0.006**	1.12	[0.74;169]	0.607	1.94	[1.31;2.88]	**0.001**

*ER^2^, Emergency room evaluation and recommendations; HR, Hazard Ratio; CI, Confidence Interval.*

*Score 0–3.

*^†^Score 4–5.*

*^‡^Score = 6. All models are adjusted by place of living, high education level, abnormal body mass index (i.e., ≥ 25 kg/m^2^) and regular physical activity; P-value significant (i.e., P < 0.05) indicated in bold.*

## Discussion

The findings show a positive association between ER^2^ risk levels and overall incidence of MNCD and AD, with a low risk level associated with low incidence and high risk level with high incidence. No association was found with incidence of non-AD.

The profile of association between ER^2^ risk levels and incidence of MNCD observed in our study is consistent with previous studies which examined incidence of adverse outcomes in older ED users (Launay et al., 2021a,b; Beauchet et al., 2022). These studies showed that a high ER^2^ risk level was associated with hospital admission and a long length of stay in ED, while a low risk level was associated with a short length of stay in ED (Launay et al., 2021a,b; Beauchet et al., 2022). The novelty of the results of the present study lies in the population, which was composed of community-dwellers rather than ED users, and its screening of a morbidity (i.e., MNCD) rather than surrogate measures like long length of stay in ED and hospital admissions.

An explanation of this positive association between ER^2^ risk levels and MNCD may be related to frailty. Frailty is defined as an individual’s health state characterized by vulnerability to stressors due to decreased physiological reserves (Blodgett et al., 2015). The ER^2^ tool may be assimilated as a short assessment of frailty using the deficit accumulation frailty model proposed by de Vries et al. (2011) and Dent et al. (2016). This model combines clinical information such as symptoms, signs, diseases and disability, and is based on the idea that a greater number of deficits indicates a higher frailty state (de Vries et al., 2011; Blodgett et al., 2015; Dent et al., 2016). We observed that the greatest association with incident MNCD was shown with the ER^2^ high risk level. Given that frailty is associated with an increased incidence of MNCD, our results confirm the rationale to integrate the ER^2^ into frailty assessments (Beauchet et al., 2022).

An operative primary prevention strategy is based on two successive steps: first, screening individuals at-risk and second, addressing their MRFs (Livingston et al., 2020; Alzheimer’s Association, 2021). MRFs are chronic morbidities like cardio-vascular risk factors (Livingston et al., 2020). Thus, an effective prevention of MNCD needs to not only consider MRFs individually, but also their accumulation and its adverse consequence. A significant adverse consequence of chronic morbidity accumulation is frailty (de Vries et al., 2011; Dent et al., 2016). Frailty is associated with an increased risk of MNCD (Chu et al., 2021). Frailty may be reversed and, thus, assimilated as a MRF of ADRD (de Vries et al., 2011; Dent et al., 2016; Chu et al., 2021). Addressing frailty might be a key intervention to prevent ADRD.

No significant association between ER^2^ risk levels and incident non-AD was reported, whereas it was with AD. This non-conclusive result for non-AD is difficult to explain. Incidence of AD and non-AD were both high (53.4 vs. 46.6%). This may be related to the various phenotypes included in non-AD dementia: non-AD dementia is caused by different brain lesions including vascular dementia, Lewy bodies dementia, or other neurodegenerative conditions.

MNCD disease-modifying treatments remain elusive, which explains the emphasis on preventive models targeting MRFs (Livingston et al., 2020; Alzheimer’s Association, 2021). A recent review of the literature shows that up to 40% of late-onset MNCD could potentially be prevented or delayed by addressing MRFs (Livingston et al., 2020). Screening people at-risk for MNCD and addressing their MRFs may thus be an effective strategy to delay or reduce incidence of MNCD. Hence, there is a need to optimize and increase accessibility to clinical risk assessment of MNCDin community-dwelling older populations. The results of the study showed that ER^2^ is simple and easy to use in clinical practice and, thus, may be incorporated as screening test for MNCD in the older population (both community-dwellers and ED visitors).

We used the term “MNCD” rather than dementia because the former is consensual and considered less stigmatizing. Dementia was replaced in the Diagnostic and Statistical Manual of Mental Disorders (DSM-5) for this reason. In fact, the word “dementia” is related to a Latin word for “mad,” or “insane.” Because of this, the introduction of the term “neurocognitive disorder” aimed to reduce the stigma associated with both the word dementia and the conditions that it refers to. When the DSM-5 was published in May 2013, the American Psychiatric Association gave a year’s grace period for the world to absorb the changes before they took effect. Acknowledging that old habits die hard, however, DSM-5 also states that use of the term “dementia” is not precluded “where that term is standard.”

The EPIDOS study design (i.e., observational population-based cohort study) and its long prospective follow-up period (7 years) are the two main strengths of the present study. However, some limitations need to be considered. First, the sample of participants was composed of only females, which limits the generalizability of findings to an older population composed of both sexes. Females have a higher risk for dementia than men (Sindi et al., 2021). It has been suggested that this difference is related to the greater life expectancy and lower cognitive reserves of females compared to males. Thus, our results cannot be generalized to address dementia risks in males. Second, participants that were cognitively impaired were excluded. We used the SPMSQ score with a threshold exclusion value ≥ 3 incorrect answers, to be sure to exclude participants with mild MNCD. This exclusion of participants with minor neurocognitive disorders, which is a risk factor for MNCD, may influenced the incidence of MNCD in our results. Third, the cognitive status of participants was determined only at the end of the 7-year follow-up. Fourth, Cox models were adjusted for baseline characteristics, but residual confounders may still be present and modify the association between ER^2^ risk stratification and incident MNCD. Additionally, comparisons between groups were not adjusted for multiple comparisons. It should also be noted that there are less people in the ER^2^ moderate risk group compared to the other groups, which may reduce the significance of the association in this group of participants. Finally, EPIDOS data were collected before 2003 and it is thus possible, though unproven, that participants’ profiles, and thus their incident risks, have changed since then.

## Conclusion

ER^2^ risk levels were associated with incident MNCD, with a high risk level predicting MNCD occurrence in EPIDOS participants. The ER^2^ tool is simple and easy to use in clinical practice. This demonstrates the potential of using ER^2^ for screening older adults at-risk of MNCD.

## Data Availability Statement

The data analyzed in this study is subject to the following licenses/restrictions: Access to EPIDOS Database can be obtain by the Contacting Corresponding author. Requests to access these datasets should be directed to the corresponding author.

## Ethics Statement

The studies involving human participants were reviewed and approved by the Research Ethics Boards (REB) of Toulouse University Hospital approved the EPIDOS protocol. The patients/participants provided their written informed consent to participate in this study.

## Author Contributions

OB and GA conceived and designed the experiments. A-MS and YR contributed to the cohort data collection. OB, CL, and GA analyzed, interpreted the data, and wrote the manuscript. OB contributed to the reagents, materials, analysis tools, and data. A-MS, YR, and JM contributed to the revision of the manuscript. All authors contributed to the article and approved the submitted version.

## Conflict of Interest

The authors declare that the research was conducted in the absence of any commercial or financial relationships that could be construed as a potential conflict of interest.

## Publisher’s Note

All claims expressed in this article are solely those of the authors and do not necessarily represent those of their affiliated organizations, or those of the publisher, the editors and the reviewers. Any product that may be evaluated in this article, or claim that may be made by its manufacturer, is not guaranteed or endorsed by the publisher.
